# Quarterly Report of the Obstetric Practice in the Philadelphia Dispensary

**Published:** 1839-10-19

**Authors:** Joseph Warrington

**Affiliations:** Accoucheur


*Quarterly Report of the Obstetric Practice in the Philadelphia Dispensary.* By JOSEPH WARRINGTON, M. D., Accoucheur.

Fifteen women have been delivered at full term. Thirteen boys and two girls have been born.

The average duration of labour in the fifteen cases was twelve hours, the extremes being three and twenty-five hours.

The average time required for the spontaneous delivery of the placenta, in thirteen cases, was twenty and a half minutes, the extremes being five and sixty minutes.

In one case, the placenta was retained within the os uteri one hour and a half, requiring finally the introduction of the hand to facilitate its delivery.

In eleven cases, in which the positions of the fœtus were ascertained, seven presented the vertex in the first position, two in the second, one in the fourth: one case presented the pelvis in the first position.

The subject of the breech presentation was still born, appearing to have been dead several days previous to delivery. Another fœtus died during a protracted labour, probably from the severe compression exerted by the uterus upon the child; there not being, apparently, a single drop of liquor amnii within the membranes.

The rest of the children have done well. All the women recovered.

Mr. Francis Drinker, a member of the obstetric class, reports the following case:

*June* 19*th*, 1839.—E. Carhart, æt. 22, subject of first pregnancy, was delivered of a female child at 4, A. M., after a labour of six hours. Vertex presentation, first position; placenta extended twenty minutes after the delivery of the child.

20*th.*—Great soreness and tenderness, under slight pressure, upon the uterine region; considerable febrile reaction; uterine contractions almost intolerant.

In consultation with Dr. Warrington, agreed to take ℥xii. of blood from the arm, give five grains of calomel in ℥ss. of castor oil, and apply fomentations of hops over the uterus.

*Evening.*—Increased fever; tenderness in uterine region not abated; medicine had not operated. Ordered enema of flaxseed tea; bled ℥viij.; gave soda powders for drink.

21*st.*—At 1, A. M., was called from bed in consequence of great jactitation, and increased fever. Bled about ℥xvi.; continued fomentations; and, at Dr. W.'s suggestion of yesterday, directed flaxseed mucilage to be injected into the vagina.

10, *A. M.*—Symptoms all mitigated directly after the last bleeding, which nearly caused syncope. Patient rapidly convalesced.

Mr. Andrew Bruce, another member of the class, reports as follows:

*Sept.* 20*th*, 1839.—A. Agen, æt. 35, (one of the patients placed under my charge by Dr. Warrington,) subject of her thirteenth pregnancy, had enjoyed usual health up to this time, when she was surprised by a very sudden discharge of blood per vaginam. This continued three days, during which it is supposed she lost six pounds of blood. She became very feeble; her pulse reduced below its usual standard of frequency and strength.

5*th*, 7 *P. M.*—I found her in labour, with strong and frequent uterine contractions. Upon examination, the os uteri was found considerably dilated, but presented a soft thick cushion, which gave the idea of an edge of the placenta. There was scarcely any sanguineous discharge at this time. The fœtus was detected with the vertex presenting in the second position. The pains being frequent and severe, the child was delivered at 9, P. M.

The patient had complained of severe cramps in the lower extremities, and the moment the head was passing through the vulva, she was seized with an hysterical convulsion. The rest of her delivery took place without her consciousness. Placenta came away in five minutes spontaneously, followed by a very free discharge of blood.

Dr. W., who had been previously sent for, now met me. We used free frictions over the uterine region, applied a graduated compress of several diapers over the hypogastrium, and pinned the bandage firmly over all. The uterus, however, becoming very flaccid, we determined upon administering five grains of powdered ergot, suspended in fluid, every half hour. I watched the patient closely during the night, and had the satisfaction to find that, after the administration of one drachm of ergot in this manner, the uterus contracted firmly, and the hæmorrhage ceased. The pulse, which, during the evening, was scarcely perceptible, and beat only sixty, had re-acted finely next morning, when the patient, who had been in a continual state of insensibility for eight hours after the convulsion, recognised surrounding objects. She promptly recovered upon the use of laxative and farinaceous diet.

The members of Dr. W.'s obstetric class are permitted to attend upon the cases which occur under his charge, in the Philadelphia Dispensary.

